# Mini review: Challenges in EEG emotion recognition

**DOI:** 10.3389/fpsyg.2023.1289816

**Published:** 2024-01-04

**Authors:** Zhihui Zhang, Josep M. Fort, Lluis Giménez Mateu

**Affiliations:** Escola Tècnica Superior d'Arquitectura de Barcelona, Universitat Politècnica de Catalunya, Barcelona, Spain

**Keywords:** EEG, emotional measurement, challenges, emotional dynamics, recognition

## Abstract

Electroencephalography (EEG) stands as a pioneering tool at the intersection of neuroscience and technology, offering unprecedented insights into human emotions. Through this comprehensive review, we explore the challenges and opportunities associated with EEG-based emotion recognition. While recent literature suggests promising high accuracy rates, these claims necessitate critical scrutiny for their authenticity and applicability. The article highlights the significant challenges in generalizing findings from a multitude of EEG devices and data sources, as well as the difficulties in data collection. Furthermore, the disparity between controlled laboratory settings and genuine emotional experiences presents a paradox within the paradigm of emotion research. We advocate for a balanced approach, emphasizing the importance of critical evaluation, methodological standardization, and acknowledging the dynamism of emotions for a more holistic understanding of the human emotional landscape.

## Introduction

In the rapidly evolving field of neuroscience and technological integration, Electroencephalography (EEG) has emerged as a pivotal tool, offering profound insights into human brain functions, especially in the study of emotional responses. Emotions, as complex amalgamations of physiological and cognitive reactions, deeply influence our daily interactions, decision-making processes, and even our experiences within architectural settings (Brunner-Sperdin et al., 2012; Nummenmaa et al., 2012; Lehman et al., 2015; Zhang et al., 2022). Emotions play a significant role in shaping human perception and interaction. This makes the study of emotional responses important in various fields, including healthcare and architecture. Understanding these responses is not just academically intriguing; it also has practical implications in these sectors.

Although EEG is increasingly favored due to its non-invasiveness and high temporal resolution, the in-depth analysis of the data it generates is particularly crucial. Numerous studies have underscored the methods of using EEG in emotional research (Chen et al., 2015; Christensen and Abdullah, 2018; Suhaimi et al., 2020; Dadebayev et al., 2021; Li et al., 2022). However, discussions around the quality of EEG emotional measurements are less prominent. While a careful review acknowledges the contributions of these studies, it also unveils oversights, particularly in addressing the intricacies and challenges of EEG data quality. For instance, Cohen et al. (2023) highlights the need for rigorous validation of EEG data in emotional research. These concerns suggest that the allure of high success rates and groundbreaking discoveries might sometimes overshadow the inherent complexities and limitations of EEG, necessitating a more critical examination of its use in emotional studies.

This article explores the complex aspects of emotion research using EEG. It critically examines the claims of high accuracy in the field and discusses the fundamental nature of emotions. The objective is to offer a comprehensive analysis that encompasses the technical challenges associated with EEG data, the impact of equipment and data source variability on application, and the paradoxes faced in conducting emotion research in controlled settings. The paper aims to contribute to a detailed and nuanced understanding of emotional science exploration.

## Methodology

To gain a comprehensive understanding of the challenges and dilemmas in EEG emotion measurement, we embarked on an in-depth literature review in August 2023, focusing our efforts on three pivotal academic databases: Web of Science, Scopus, and ProQuest. These databases were chosen for their extensive coverage in neuroscience and psychological research. Using the keyword combination of “EEG” and “emotion”, we aimed to collate core research papers central to our topic. This initial search spanning the years 2022 to 2023 yielded a significant count of 3,741 articles. Recognizing the voluminous nature of our initial pool and the need for stringent quality control, we employed the MMAT (Mixed Methods Appraisal Tool) (Hong et al., 2018) to assess and sieve these articles. The MMAT, renowned for its capability to critically assess mixed methods research, served as a foundational filter in our process.

**Figure 1:**
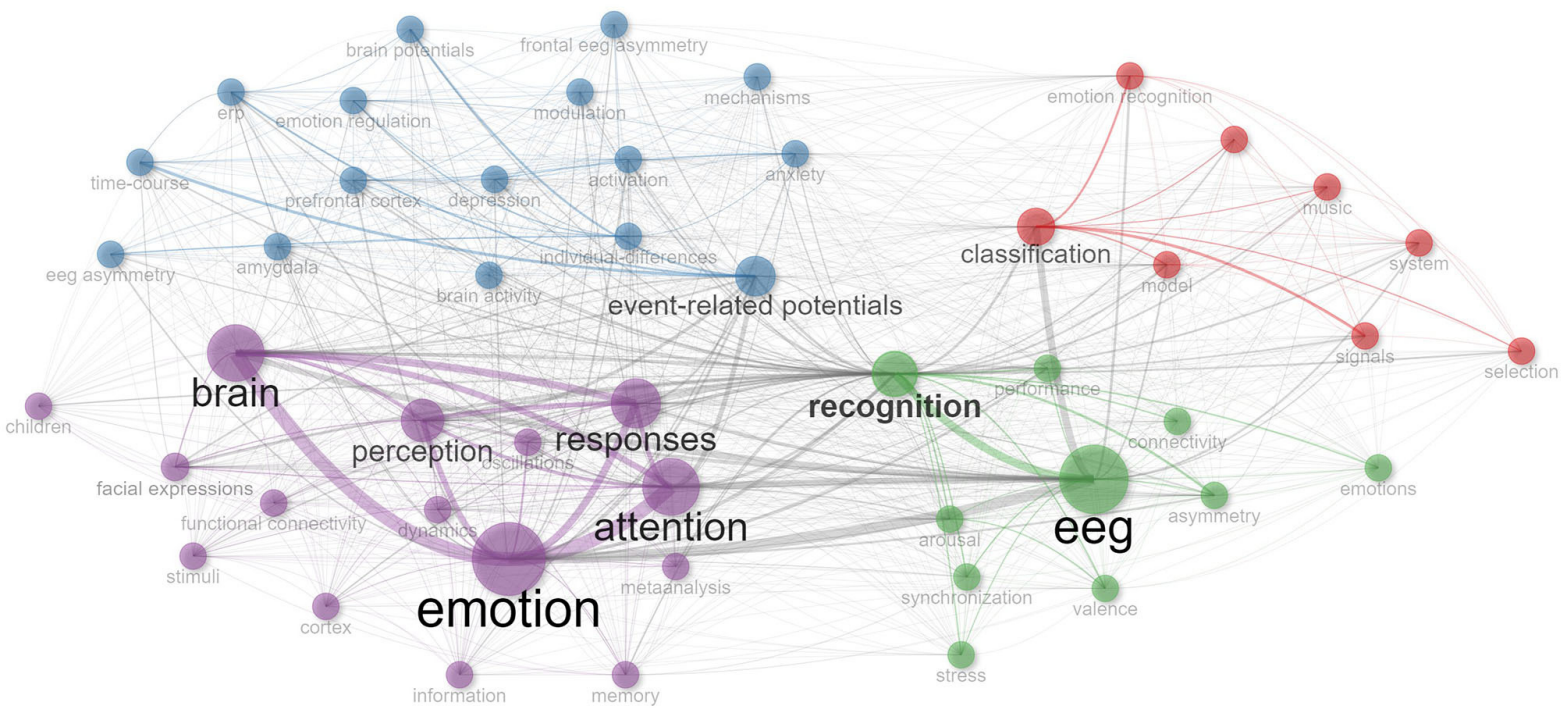
Visual representation of EEG emotion research: co-citation and keyword relationships.

The subsequent phase of our methodology centered on refining our selection based on the robustness of experimental results. We pivoted our attention toward articles that reported an emotion recognition accuracy rate exceeding 90%, a threshold we deemed crucial as it represents a benchmark in EEG-based emotion recognition that suggests robust and replicable findings. This rigorous criterion distilled our initial list down to 22 quintessential articles poised for an in-depth analysis. To further elevate the quality of our review and to draw comprehensive insights, we employed the bibliometrix packages in R studio for data visualization analysis (Aria and Cuccurullo, 2017). This sophisticated analytical tool enabled us to intricately map co-citation relationships, track research keywords, and intuitively navigate the intricate web of research relationships and emergent themes in the domain, all of which is exemplified in Figure 1.

## The reality behind high EEG accuracy rates

Recent EEG-based emotion recognition research claims strikingly high accuracy rates of 90–99% (see Table 1). These figures are particularly remarkable when juxtaposed with the 75–80% accuracy rate achieved by current facial emotion recognition technology (Naga et al., 2023). However, the majority of these EEG studies employ binary (Valence, Arousal) or ternary (Positive, Negative, Neutral) emotional models, which inherently simplify the classification task and can lead to inflated accuracy rates. For instance, Zhong et al. (2019) reported a drop in accuracy from 79.14–91.67% to 68.26–80.14% when their binary model of emotion was expanded to include four dimensions. This is particularly telling considering that standard models of basic emotions are often based on six or seven categories, suggesting that a more nuanced approach might yield significantly different accuracy rates.

**Table 1:** Recent EEG emotion recognition studies with an accuracy rate exceeding 90%.

**References**	**Data set**	**Model abbreviation**	**Model description**	**Signal processing**	**Emotion type**	**Dependence accuracy**	**Independence accuracy**	**Dependence strategy**	**Independence strategy**
Li et al. (2018)	SEED	BiDANN	Bi-hemispheres domain adversarial neural network	1D to 2D	PNN	86.15–96.89%	74.52–91.04%	TT: 9–6	15-fold C-V
Yang et al. (2018)	DEAP Koelstra et al. (2011)	CFCNN	Channel-frequency convolutional neural network	1D to 2D	VA	90.80–91.03%	Null	10-fold C-V	Null
Ma et al. (2019)	DEAP	Res-LSTM	Multimodal residual LSTM classifier	Downsampling Filtering	VA	92.87–92.30%	Null	5-fold C-V	Null
Li et al. (2022)	SEED	LSTM-based	Hierarchical spatio-temporal neural network model based on LSTM	Filtering normalization	PNN	89.05–95.48%	79.73–90.05%	TT: 9-6	15-fold C-V
Zhang et al. (2019)	SEED	Riemannian Network	Riemannian Network	Segmentation	PNN	86.40–91.55%	Null	10-fold C-V	Null
Cho and Hwang (2020)	DEAP	3D-CNN	2 types of 3D-CNN models	Segmentation	VA	99.74–99.73%	Null	5-fold C-V	Null
Zhong et al. (2019)	SEED	RGNN	Regularized GNN model	Filtering Normalization	NSFH (VA)	71.85–91.92% (90.04–97.60%)	68.26–80.14% (79.14–91.67%)	15-fold C-V	15-fold C-V
Zhang et al. (2020)	SEED, MPED Song et al. (2019)	VPR	Heuristic variational pathway reasoning	Filtering	PNN	93.21–95.67% (SD) 61.61–80.95% (MD)	Null	TT: 9-6	Null
Cui et al. (2020)	DEAP, DREAMER Katsigiannis and Ramzan (2017)	RACNN	End-to-end regional-asymmetric CNN	Segmentation	VA	96.65–97.11% (DP) 95.55–97.01% (DR)	Null	10-fold C-V	Null
Wang et al. (2020)	SEED, DEAP	EFDMs+STFT	Electrode-frequency distribution maps with short-time	Segmentation	PNN	88–93% (SD) 79–86% (DP)	Null	TT: 9–6 (SD) TT: 4–1 (DP)	Null
Tao et al. (2023)	DEAP, DREAMER	ACRNN	Attention-based convolutional recurrent neural network	Segmentation	VA	93.38–93.72% (DP) 97.78–98.23% (DR)	Null	10-fold C-V	Null
Luo et al. (2020)	SEED, DEAP	VAE + GAN	Variational autoencoder and generative adversarial network	Filtering	PNN	87.5–93.5%	Null	5-fold C-V	Null
Cheng et al. (2021)	DEAP, DREAMER	GcForest	Multi-grained cascade Forest model	Segmentation	VA	97.69–97.53% (DP) 89.03–90.41% (DR)	Null	10-fold C-V	Null
Liu et al. (2020)	DEAP, DREAMER	MLF-CapsNe	Effective multi-level features guided capsule network	1D to 2D	VA	97.97–98.31% (DP) 94.59–95.26% (DR)	Null	10-fold C-V	Null
He et al. (2020)	LabEdata, DEAP	Firefly optimization	A novel firefly integrated optimization algorithm	Filtering	VA	84.21–96.77% (DP) 91–99% (LabEdata)	Null	TT: 3-1	Null
Huang et al. (2021)	DEAP	BiDCNN	Bi-hemisphere discrepancy convolutional neural network	Segmentation	VA	94.38–94.72%	68.14–63.94%	10-fold C-V	32-fold C-V
Yin et al. (2021)	DEAP	ECGGCNN	GCNN-LSTM hybrid model	1D to 2D	VA	90.45–90.60	84.81–85.27%	5-fold C-V	3*5-fold C-V
Zhang and Etemad (2023)	SEED	LSTM-Capsule	Long short-term memory capsule network	Filtering Segmentation	VA	0.9107 ± 0.0763	Null	5-fold C-V	Null
Fdez et al. (2021)	SEED	CNN	Convolutional neural network	Filtering Normalization	PN\PNN	Null	91.6% (PN) 79.6% (PNN)	Null	15-fold C-V
Zhu et al. (2022)	SEED	ECN-AF	Emotion classification network based on attention fusion	Filtering Segmentation	PNN	95.87–96.45%	Null	5-fold C-V	Null

PNN, positive, negative, neutral; PN, positive, negative; VA, valence, arousal; C-V, cross-validation; TT, train:test; SD, SEED; MD, MPED; DP, DEAP; DR, DREAMER; Null, not mentioned in the article.

In discussions of EEG-based emotion recognition accuracy, it becomes evident that models relying on subject-dependent data often report higher accuracy rates. This is because when a model is trained and tested on data from the same participant, it can more effectively capture specific characteristics of that individual, leading to increased accuracy. For instance, the BiDANN model by Li et al. (2022) demonstrated an accuracy range of 86.15–96.89% on the SEED dataset (Zheng and Lu, 2015) in a subject-dependent scenario, while the accuracy dropped to 74.52–91.04% in subject-independent settings. Similarly, the BiDCNN model by Huang et al. (2021) achieved emotion recognition accuracy rates of 94.38–94.72% using subject-dependent strategies, which significantly declined to 68.14–63.94% under subject-independent conditions. This phenomenon highlights the substantial reduction in overall accuracy rates when shifting from subject-dependent to subject-independent validation strategies. Independent subject validation poses a greater challenge as it requires the model to generalize to data from unseen participants. This generalization often reveals limitations of the model, as it must capture broader and more universal emotional features rather than merely adapting to specific training data. Therefore, when assessing the true performance of EEG emotion recognition technologies, special attention should be paid to the accuracy rates reported under subject-independent conditions.

The choice of *k*-value in cross-validation significantly impacts the model's generalization ability and accuracy. Although most studies employ 10- or 15-fold cross-validation, which helps to reduce the risk of overfitting to specific data splits, there are situations, such as the use of 5-fold cross-validation, especially when data splits are not sufficiently random or the dataset size is small, where the model may still face the risks of overfitting and artificially inflated accuracy rates. Moreover, the use of windowing and segmentation as data augmentation strategies warrants attention in these studies. When data is augmented through windowing and segmentation, the model may become overly proficient at recognizing repetitive or similar data segments, leading to seemingly improved performance during testing. While this data augmentation strategy can enhance model performance in some cases, it may also lead to poor generalization on new, unseen data, thereby significantly compromising accuracy in practical applications.

Therefore, while it seems that EEG emotion analysis has made significant progress due to deep learning, the high accuracy rates often overshadow the selective presentation of results by researchers under publication pressures.

## The challenge of application

In the field of EEG-based emotion recognition, although there are technical challenges such as physiological disturbances to brain signals from sources like body movement, muscle electrical interference, eye movements, and heartbeats (Fatourechi et al., 2004), the greater challenge lies in applying these techniques to practical scenarios. Unlike facial emotion recognition, which primarily uses images, the data sources for EEG-based emotion detection are more intricate and varied. A notable observation is that most EEG-based emotion recognition studies rely on pre-existing datasets [such as SEED, DEAP (Koelstra et al., 2011), DREAMER (Katsigiannis and Ramzan, 2017)] rather than collecting data independently (Kumari et al., 2022). Furthermore, there seems to be a scarcity of research in applying self-designed and trained models to real-world emotion measurement experiments. Several reasons contribute to this scenario:

- **Lack of standardization in EEG devices:** While image-based facial recognition technologies can often normalize variations through advanced processing, EEG data faces greater challenges in standardization due to the lack of consistent device standards. EEG products can be classified based on the number of electrodes: more than 32 electrodes (high-density systems), 8–32 electrodes (medium-density systems), and fewer than eight electrodes (low-density systems). Additionally, the type of electrode—be it wet, dry, or specialized—adds to the complexity (Hernandez-Pavon et al., 2023). While wet electrodes using conductive gels or saline might offer better signal quality, they can be more cumbersome and intrusive, potentially affecting the emotional state of the participant. On the other hand, consumer-grade EEG devices, typically designed more for entertainment than research, often use fewer electrodes (1–16) and may not be suitable for rigorous scientific study.
- **Challenges in data collection:** Collecting EEG data presents a unique set of challenges, primarily due to the need for specialized knowledge and high-quality capture techniques (Boudewyn et al., 2023). Unlike more accessible fields like video tracking or facial emotion recognition, EEG data collection demands specialized expertise and sophisticated equipment. Here, “regular researchers” refers to those who might not specialize in neuroscientific methods or lack access to advanced EEG data collection facilities. These general researchers, typically found in broader disciplines without a focus on neurology or bioengineering, often face challenges in acquiring and processing EEG data due to limited facilities or expertise. Such limitations in data gathering can impede the development of robust machine learning models, affecting their generalizability and effectiveness.

Given these challenges, it's clear that emotion recognition based on EEG is not just a matter of recognition technique but also involves the availability of data and equipment. The fragmented nature of data sources, combined with the diversity of EEG devices and the complexities of data collection, poses significant obstacles to the advancement and generalization of EEG-based emotion recognition research.

## The paradox of emotional research

The disconnect between laboratory environments and genuine emotional experiences is a key issue in emotion studies. Laboratory settings may be too rigid and artificial, failing to reflect real emotional experiences in daily life (Russell, 1994). Limitations of laboratory-based emotional research include the simulation of real-life scenarios and individual differences in emotional experience (Rottenberg and Gross, 2007). We need to balance laboratory setups and real-life emotional experiences (Healey et al., 2010).

Experimental equipment may interfere with participants' natural emotional experiences. Advanced devices like virtual reality present potential value and challenges in emotional research, including potential interference with participant emotions (Bohil et al., 2011). Especially with EEG devices, the requirement for participants to remain still to avoid movement interference may restrict natural emotional expression (Wilhelm and Grossman, 2010).

The mismatch between the dynamism of emotions and the fixed nature of experimental design may limit research accuracy and relevance. Emotions' fluidity and change have been emphasized as core parts of research (Kuppens et al., 2010). Time scale issues in emotional research, especially in capturing dynamic emotional changes, have been explored (Davidson, 2010). The complexity of emotional research is revealed with the tension between dynamism and stillness, suggesting EEG devices might be unsuitable as emotional measuring tools.

## Discussion

The use of EEG in emotion recognition research presents a host of possibilities and pitfalls. This confluence of neuroscience and technological integration represents a frontier in our understanding of human emotional processes, yet as our review suggests, researchers need to tread cautiously. The lure of high accuracy rates, the challenges of generalization, and the inherent paradoxes of emotional research have proven to be formidable challenges.

There's no doubt that the temporal resolution of EEG offers a granular view of neural processes as emotions unfold (MacNamara et al., 2022). However, as our review suggests, the translation from granular neural data to accurate emotional recognition is far from straightforward. A primary concern is the reported accuracy rates, which in some instances seem too good to be true. These astonishingly high rates challenge our understanding of the complexities of both EEG data and the nuanced nature of human emotion. High accuracy rates, when not critically examined, can lead to a false sense of progress in the field.

The challenge of application further exacerbates these concerns. Emotion, by its nature, is influenced by myriad factors, from the immediate environment to an individual's past experiences (Jani and Han, 2015). Thus, relying on standard datasets, while practical, may not capture the full gamut of human emotion. The diversity in EEG devices, data sources, and collection methodologies can introduce variability that complicates generalization. Additionally, as mentioned, the lack of standardization in EEG devices makes the reproducibility of research findings a formidable challenge (Keil et al., 2014).

Furthermore, the paradoxical nature of emotional research, especially the gulf between lab conditions and real-world emotional experiences, cannot be understated. Emotions are not static entities to be captured in controlled environments but are dynamically intertwined with our ever-changing contexts (Barrett et al., 2006; Wilhelm and Grossman, 2010). The very act of measuring emotion in a lab setting might alter the nature of the emotion itself, akin to the observer effect in physics (Russell, 2003). The complexities associated with dynamic emotional changes underline the importance of methodological flexibility and the need for tools that can capture the rich tapestry of human emotions.

## Conclusion

In this comprehensive review, we have shed light on the intricacies of EEG-based emotion recognition. While the allure of high accuracy rates in EEG emotion research paints an optimistic picture, it is essential to approach these claims with cautious optimism. The challenges in generalizing these methods, the inherent discrepancies in laboratory environments, and the dynamic nature of emotions are significant cautions for those eager to adopt EEG in emotion recognition. We must recognize that although EEG holds great potential as a tool in emotion research, its ability to fully understand human emotions is not yet perfect and requires further development and refinement.

## Limitations, implications, and further directions of research

In our comprehensive review of EEG-based emotion recognition, we identified several key limitations and implications for the broader scientific community. Our review, concentrated on literature from 2018 to 2022, may have missed broader developments in the field. Our focus on high-accuracy studies could also have overshadowed valuable insights from moderate or lower-accuracy research. Further, relying on databases like Web of Science, Scopus, and ProQuest could have led us to overlook significant work housed in niche repositories. The highlighted accuracies in studies emphasize the need for a critical evaluation regarding methodological rigor and the clear divergence between laboratory and real-world settings, calling for more standardized and applicable tools and methodologies. Moving forward, there's an evident need to expand the scope of the literature review, hone in on the most effective methodologies, integrate multiple biometric tools for a more comprehensive emotion assessment, prioritize studies with higher ecological validity, and deeply investigate the influence of diverse emotion theories on EEG data interpretation. As we delve deeper into this domain, embracing these considerations could lead to a more nuanced understanding of the human emotional landscape through EEG research.

## Author contributions

ZZ: Conceptualization, Methodology, Visualization, Writing – original draft. JF: Conceptualization, Data curation, Writing – review & editing. LG: Conceptualization, Methodology, Writing – review & editing.

## References

[B1] Aria M. Cuccurullo C. (2017). bibliometrix: an r-tool for comprehensive science mapping analysis. J. Informetr. 11, 959–975. 10.1016/j.joi.2017.08.007

[B2] Barrett L. F. Mesquita B. Ochsner K. N. Gross J. J. (2006). The experience of emotion. Annu. Rev. Psychol. 58, 373–403. 10.1146/annurev.psych.58.110405.085709 PMC1934613 17002554

[B3] Bohil C. J. Alicea B. Biocca F. A. (2011). Virtual reality in neuroscience research and therapy. Nat. Rev. Neurosci. 12, 752–762. 10.1038/nrn3122 22048061

[B4] Boudewyn M. A. Erickson M. A. Winsler K. Ragland J. D. Yonelinas A. Frank M.. (2023). Managing eeg studies: how to prepare and what to do once data collection has begun. Psychophysiology 60, e14365. 10.1111/psyp.14365 37314113 PMC11276027

[B5] Brunner-Sperdin A. Peters M. Strobl A. (2012). It is all about the emotional state: managing tourists' experiences. Int. J. Hosp. Manag. 31, 23–30. 10.1016/j.ijhm.2011.03.004

[B6] Chen J. Hu B. Moore P. Zhang X. Ma X. (2015). Electroencephalogram-based emotion assessment system using ontology and data mining techniques. Appl. Soft Comput. 30, 663–674. 10.1016/j.asoc.2015.01.007

[B7] Cheng J. Chen M. Li C. Liu Y. Song R. Liu A.. (2021). Emotion recognition from multi-channel eeg via deep forest. IEEE J. Biomed. Health Inf. 25, 453–464. 10.1109/JBHI.2020.2995767 32750905

[B8] Cho J. Hwang H. (2020). Spatio-temporal representation of an electoencephalogram for emotion recognition using a three-dimensional convolutional neural network. Sensors 20, 3491. 10.3390/s20123491 32575708 PMC7349167

[B9] Christensen L. R. Abdullah M. A. (2018). “EEG emotion detection review,” in 2018 IEEE Conference on Computational Intelligence in Bioinformatics and Computational Biology (CIBCB) (St. Louis, MO), 1–7. 10.1109/CIBCB.2018.8404976

[B10] Cohen S. Zantvoord J. Wezenberg B. Daams J. Bockting C. Denys D.. (2023). Electroencephalography for predicting antidepressant treatment success: a systematic review and meta-analysis. J. Affect. Disord. 321, 201–207. 10.1016/j.jad.2022.10.042 36341804

[B11] Cui H. Liu A. Zhang X. Chen X. Wang K. Chen X. (2020). Eeg-based emotion recognition using an end-to-end regional-asymmetric convolutional neural network. Knowl. Based Syst. 205, 106243. 10.1016/j.knosys.2020.106243

[B12] Dadebayev D. Goh W. W. Tan E. X. (2021). Eeg-based emotion recognition: review of commercial eeg devices and machine learning techniques. J. King Saud Univ. Comput. Inf. Sci. 34, 4385–4401. 10.1016/j.jksuci.2021.03.009

[B13] Davidson R. J. (2010). Affective style and affective disorders: perspectives from affective neuroscience. APA PsycNet 12, 307–330. 10.1080/026999398379628

[B14] Fatourechi M. Mason S. G. Birch G. E. Ward R. K. (2004). “A wavelet-based approach for the extraction of event related potentials from eeg,” in 2004 IEEE International Conference on Acoustics, Speech, and Signal Processing (Montreal, QC: IEEE), ii–737. 10.1109/ICASSP.2004.1326363

[B15] Fdez J. Guttenberg N. Witkowski O. Pasquali A. (2021). Cross-subject eeg-based emotion recognition through neural networks with stratified normalization. Front. Neurosci. 15, 626277. 10.3389/fnins.2021.626277 33613187 PMC7888301

[B16] He H. Tan Y. Ying J. Zhang W. (2020). Strengthen eeg-based emotion recognition using firefly integrated optimization algorithm. Appl. Soft Comput. 94, 106426. 10.1016/j.asoc.2020.106426

[B17] Healey J. Nachman L. Subramanian S. Shahabdeen J. Morris M. (2010). “Out of the lab and into the fray: towards modeling emotion in everyday life,” in Pervasive Computing. Pervasive 2010. Lecture Notes in Computer Science, Vol. 6030, eds Floréen P. Krüger A. Spasojevic M. (Berlin; Heidelberg: Springer). 10.1007/978-3-642-12654-3_10

[B18] Hernandez-Pavon J. C. Veniero D. Bergmann T. O. Belardinelli P. Bortoletto M. Casarotto S.. (2023). Tms combined with eeg: Recommendations and open issues for data collection and analysis. Brain Stimul. 16, 567–593. 10.1016/j.brs.2023.02.009 36828303

[B19] Hong Q. N. Fàbregues S. Bartlett G. Boardman F. Cargo M. Dagenais P.. (2018). The mixed methods appraisal tool (mmat) version 2018 for information professionals and researchers. Educ. Inf. 34, 285–291. 10.3233/EFI-180221

[B20] Huang D. Chen S. Liu C. Zheng L. Tian Z. Jiang D. (2021). Differences first in asymmetric brain: a bi-hemisphere discrepancy convolutional neural network for eeg emotion recognition. Neurocomputing 448, 140–151. 10.1016/j.neucom.2021.03.105

[B21] Jani D. Han H. (2015). Influence of environmental stimuli on hotel customer emotional loyalty response: Testing the moderating effect of the big five personality factors. Int. J. Hosp. Manag. 44, 48–57. 10.1016/j.ijhm.2014.10.006

[B22] Katsigiannis S. Ramzan N. (2017). Dreamer: a database for emotion recognition through eeg and ecg signals from wireless low-cost off-the-shelf devices. IEEE J. Biomed. Health Inf. 22, 98–107. 10.1109/JBHI.2017.2688239 28368836

[B23] Keil A. Debener S. Gratton G. Junghöfer M. Kappenman E. S. Luck S. J.. (2014). Committee report: Publication guidelines and recommendations for studies using electroencephalography and magnetoencephalography. Psychophysiology 51, 1–21. 10.1111/psyp.12147 24147581

[B24] Koelstra S. Muhl C. Soleymani M. Lee J.-S. Yazdani A. Ebrahimi T.. (2011). Deap: a database for emotion analysis; using physiological signals. IEEE Transact. Affect. Comp. 3, 18–31. 10.1109/T-AFFC.2011.15

[B25] Kumari N. Anwar S. Bhattacharjee V. (2022). Time series-dependent feature of eeg signals for improved visually evoked emotion classification using emotioncapsnet. Neural Comp. Appl. 34, 13291–13303. 10.1007/s00521-022-06942-x

[B26] Kuppens P. Oravecz Z. Tuerlinckx F. (2010). Feelings change: accounting for individual differences in the temporal dynamics of affect. J. Pers. Soc. Psychol. 99, 1042–1060. 10.1037/a0020962 20853980

[B27] Lehman B. J. Cane A. C. Tallon S. J. Smith S. F. (2015). Physiological and emotional responses to subjective social evaluative threat in daily life. Anxiety Stress Coping 28, 321–339. 10.1080/10615806.2014.968563 25264711

[B28] Li Y. Zheng W. Cui Z. Zhang T. Zong Y. (2018). “A novel neural network model based on cerebral hemispheric asymmetry for eeg emotion recognition,” in Proceedings of the Twenty-Seventh International Joint Conference on Artificial Intelligence, IJCAI-18 (Stockholm: International Joint Conferences on Artificial Intelligence Organization), 1561–1567. 10.24963/ijcai.2018/216

[B29] Li Y. Zheng W. Wang L. Zong Y. Cui Z. (2022). From regional to global brain: a novel hierarchical spatial-temporal neural network model for eeg emotion recognition. IEEE Transact. Affect. Comp. 13, 568–578. 10.1109/TAFFC.2019.2922912

[B30] Liu Y. Ding Y. Li C. Cheng J. Song R. Wan F.. (2020). Multi-channel eeg-based emotion recognition via a multi-level features guided capsule network. Comput. Biol. Med. 123, 103927. 10.1016/j.compbiomed.2020.103927 32768036

[B31] Luo Y. Zhu L. Z. Wan Z. Y. Lu B. L. (2020). Data augmentation for enhancing eeg-based emotion recognition with deep generative models. J. Neural Eng. 17, 056021. 10.1088/1741-2552/abb580 33052888

[B32] Ma J. Tang H. Zheng W.-L. Lu B.-L. (2019). “Emotion recognition using multimodal residual LSTM network,” in Proceedings of the 27th ACM International Conference on Multimedia (MM '19) (Nbookew York, NY: Association for Computing Machinery), 176–183. 10.1145/3343031.3350871

[B33] MacNamara A. Joyner K. Klawohn J. (2022). Event-related potential studies of emotion regulation: a review of recent progress and future directions. Int. J. Psychophysiol. 176, 73–88. 10.1016/j.ijpsycho.2022.03.008 35346736 PMC9081270

[B34] Naga P. Marri S. D. Borreo R. (2023). Facial emotion recognition methods, datasets and technologies: a literature survey. Materials Today 80, 2824–2828. 10.1016/j.matpr.2021.07.046

[B35] Nummenmaa L. Glerean E. Viinikainen M. Jääskeläinen I. P. Hari R. Sams M. (2012). Emotions promote social interaction by synchronizing brain activity across individuals. Proc. Nat. Acad. Sci. U. S. A. 109, 9599–9604. 10.1073/pnas.1206095109 22623534 PMC3386135

[B36] Rottenberg J. Gross J. J. (2007). Emotion and emotion regulation: a map for psychotherapy researchers. Clin. Psychol.: Sci. Pract. 14, 323–328. 10.1111/j.1468-2850.2007.00093.x

[B37] Russell J. A. (1994). Is there universal recognition of emotion from facial expression? A review of the cross-cultural studies. Psychol. Bull. 115, 102–141. 10.1037/0033-2909.115.1.102 8202574

[B38] Russell J. A. (2003). Core affect and the psychological construction of emotion. Psychol. Rev. 110, 145–172. 10.1037/0033-295X.110.1.145 12529060

[B39] Song T. Zheng W. Lu C. Zong Y. Zhang X. Cui Z. (2019). Mped: a multi-modal physiological emotion database for discrete emotion recognition. IEEE Access 7, 12177–12191. 10.1109/ACCESS.2019.2891579

[B40] Suhaimi N. S. Mountstephens J. Teo J. (2020). Eeg-based emotion recognition: a state-of-the-art review of current trends and opportunities. Comput. Intell. Neurosci. 2020, 8875426. 10.1155/2020/8875426 33014031 PMC7516734

[B41] Tao W. Li C. Song R. Cheng J. Liu Y. Wan F.. (2023). Eeg-based emotion recognition via channel-wise attention and self attention. IEEE Transact. Affect. Comp. 14, 382–393. 10.1109/TAFFC.2020.3025777

[B42] Wang F. Wu S. Zhang W. Xu Z. Zhang Y. Wu C.. (2020). Emotion recognition with convolutional neural network and eeg-based efdms. Neuropsychologia 146, 107506. 10.1016/j.neuropsychologia.2020.107506 32497532

[B43] Wilhelm F. H. Grossman P. (2010). Emotions beyond the laboratory: theoretical fundaments, study design, and analytic strategies for advanced ambulatory assessment. Biol. Psychol. 84, 552–569. 10.1016/j.biopsycho.2010.01.017 20132861

[B44] Yang Y. Wu Q. Qiu M. Wang Y. Chen X. (2018). “Emotion recognition from multi-channel eeg through parallel convolutional recurrent neural network,” in 2018 International Joint Conference on Neural Networks (IJCNN) (Rio de Janeiro: IEEE). 10.1109/IJCNN.2018.8489331

[B45] Yin Y. Zheng X. Hu B. Zhang Y. Cui X. (2021). Eeg emotion recognition using fusion model of graph convolutional neural networks and lstm. Appl. Soft Comput. 100, 106954. 10.1016/j.asoc.2020.106954

[B46] Zhang G. Etemad A. (2023). Distilling eeg representations via capsules for affective computing. Pattern Recognit. Lett. 171, 99–105. 10.1016/j.patrec.2023.05.011

[B47] Zhang T. Cui Z. Xu C. Zheng W. Yang J. (2020). Variational pathway reasoning for eeg emotion recognition. Proc. AAAI Conf. Artif. Intell. 34, 2709–2716. 10.1609/aaai.v34i03.5657

[B48] Zhang T. Zheng W. Cui Z. Zong Y. Li Y. (2019). Spatial-temporal recurrent neural network for emotion recognition. IEEE Trans. Cybern. 49, 939–947. 10.1109/TCYB.2017.2788081 29994572

[B49] Zhang Z. Mir J. M. F. Mateu L. G. (2022). The effects of white versus coloured light in waiting rooms on people's emotions. Buildings 12, 1356. 10.3390/buildings12091356

[B50] Zheng W.-L. Lu B.-L. (2015). Investigating critical frequency bands and channels for eeg-based emotion recognition with deep neural networks. IEEE Trans. Auton. Ment. Dev. 7, 162–175. 10.1109/TAMD.2015.2431497

[B51] Zhong P. Wang D. Miao C. (2019). Eeg-based emotion recognition using regularized graph neural networks. IEEE Transact. Affect. Comp. 13, 1290–1301. 10.1109/TAFFC.2020.2994159

[B52] Zhu X. Rong W. Zhao L. He Z. Yang Q. Sun J.. (2022). Eeg emotion classification network based on attention fusion of multi-channel band features. Sensors 22, 5252. 10.3390/s22145252 35890933 PMC9318779

